# A functional module-based exploration between inflammation and cancer in esophagus

**DOI:** 10.1038/srep15340

**Published:** 2015-10-22

**Authors:** Nannan Liu, Chunhua Li, Yan Huang, Ying Yi, Wanlan Bo, Chunmiao Li, Yue Li, Yongfei Hu, Kongning Li, Hong Wang, Liwei Zhuang, Huihui Fan, Dong Wang

**Affiliations:** 1 College of Bioinformatics Science and Technology, Harbin Medical University, Harbin, Heilongjiang 150086, China; 2 Department of Network Information Center, Harbin Medical University, Harbin, Heilongjiang 150086, China; 3 The Fourth Affiliated Hospital, Harbin Medical University, Harbin, Heilongjiang 150001, China

## Abstract

Inflammation contributing to the underlying progression of diverse human cancers has been generally appreciated, however, explorations into the molecular links between inflammation and cancer in esophagus are still at its early stage. In our study, we presented a functional module-based approach, in combination with multiple data resource (gene expression, protein-protein interactions (PPI), transcriptional and post-transcriptional regulations) to decipher the underlying links. Via mapping differentially expressed disease genes, functional disease modules were identified. As indicated, those common genes and interactions tended to play important roles in linking inflammation and cancer. Based on crosstalk analysis, we demonstrated that, although most disease genes were not shared by both kinds of modules, they might act through participating in the same or similar functions to complete the molecular links. Additionally, we applied pivot analysis to extract significant regulators for per significant crosstalk module pair. As shown, pivot regulators might manipulate vital parts of the module subnetworks, and then work together to bridge inflammation and cancer in esophagus. Collectively, based on our functional module analysis, we demonstrated that shared genes or interactions, significant crosstalk modules, and those significant pivot regulators were served as different functional parts underlying the molecular links between inflammation and cancer in esophagus.

Epidemiological observations have demonstrated that chronic inflammation predisposes individuals to diverse types of cancers^1^. Corresponding inflammatory responses and infections are estimated to link to 15 ~ 20% of all deaths from cancer worldwide^2^. Specifically, in esophagus, chronic epithelial irritation-caused inflammatory processes involved in up to 90% of esophageal cancer patients^3^. Though the close link between inflammation and cancer has been well-studied in most cancers, underlying explorations on pathogenesis in esophagus are still lacking.

Esophagus cancer, as an aggressive gastrointestinal cancer, is the fifth leading cause of cancer-related death in the world^4^, with an overall five-year survival rate around 10%^5^. Previous efforts have been mostly dedicated to identify individual genes or gene pathways in esophageal cancer. Li *et al.* related gene ECRG1 with the development of esophageal cancer, especially in those patients with a long history of smoking^6^. Dulak *et al.* applied whole-exome sequencing to large cohort of tumor-normal pairs in esophagus, and then identified recurrent driver events underlying the cancer^7^. Subsequent functional analyses suggested that the potential activation of RAC1 pathway, indicated by the increasing cellular invasion after mutation in gene ELMO1, might contribute to the tumorigenesis of esophagus^7^. Besides, several other researches have focused on the studies of oesophagitis. Via up-regulation of gene KLF4, Vega *et al.* proofed that the inhibition of notch pathway could promote the differentiation of esophageal cells towards Barrett-like metaplasia, which is a common cause of esophageal diseases, such as cancer^8^. Nevertheless, few studies so far have explored the underlying molecular links between inflammation and cancer in esophagus.

Here, we presented a functional module-based approach to explore how inflammation is linked with cancer in esophagus. According to differentially expressed genes (DEGs) identified in oesophagitis and esophageal cancer, we mapped those DEGs to protein-protein interaction (PPI) network, and then extracted functional inflammation- and cancer-related modules, respectively. We observed that, common module DEGs and interactions tended to play important roles in linking inflammation and cancer. Besides, based on module crosstalk analysis, we examined two module subnetworks, one with inflammation module surrounded by cancer modules, and the other with cancer module surrounded by inflammation modules. In our results, we showed that, even though those DEGs were not shared by different inflammation and cancer modules, they might involve in the same or similar functions employed by inflammation or cancer to complete their molecular links. Additionally, based on transcriptional and post-transcriptional regulations, we identified pivot regulators for each module pair of inflammation and cancer. Functional analysis indicated that, they might function through regulating key module DEGs to bridge inflammation and cancer. Collectively, our functional module-based strategy, not only help to explore those underlying molecular mechanisms between inflammation and cancer in esophagus, but also provide a rich resource for biologists to further design their researches.

## Results

### Identification of functional inflammation- and cancer-related modules

Based on normalized gene expression profiles of esophagitis and esophageal cancer from GEO^9^ database (Table 1), we applied SAM^10^ to identify differentially expressed genes (DEGs). In total, we obtained 6132 inflammation-DEGs, with 1854 up-regulated and 4278 down-regulated. As for cancer-DEGs, we acquired 4533 DEGs in total, with 2796 showing up-regulations and 1737 down-regulations. Based on validated cancer genes, we collected from database OMIM^11^ and DisGeNET^12^ which organized the data of CTD^13^, GAD^14^, RGD^13^, LHGDN^15^ and BeFree^16^ (Supplementary Table 1), we showed that 38 validated genes were identified as cancer-DEGs in our data sets, which therefore demonstrated that our identified DEGs were suitable to represent disease conditions.

**Figure 1:**
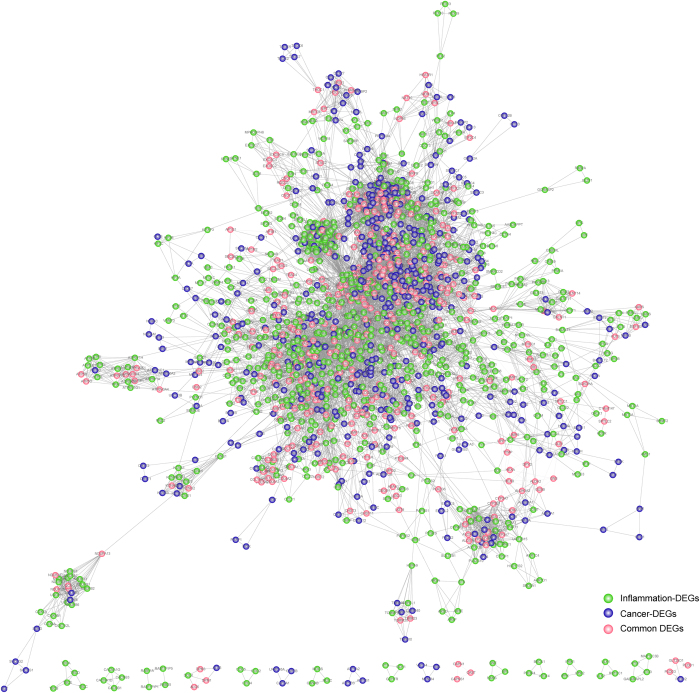
The whole inflammation- and cancer-related functional module network. Inflammation and cancer modules were extracted using MCODE program, after mapping differentially expressed genes (DEGs) to human PPI network, separately. Nodes are colored as inflammation (green ball), cancer (blue ball) or common DEGs (red ball).

In order to identify functional clustered inflammation- and cancer-DEGs, we applied MCODE^17^ to compute modules underlying inflammation and cancer, via mapping those inflammation- and cancer-DEGs to PPI^18^ network, separately. In our results, we obtained 118 inflammation- (Supplementary Table 2) and 110 cancer-related (Supplementary Table 3) functional modules, respectively, which formed a maximal connected component including 1629 nodes and 15887 edges (Fig. 1).

As indicated, inflammation- and cancer-DEGs tended to cluster tightly together inside modules, respectively. We also observed common genes serving as both inflammation- and cancer-DEGs, and common interactions emerging between two kinds of modules. For example, a module containing gene DSG2, PKP2 and DSC2, is served as both inflammation and cancer module. As previously reported, desmosome component DSG2 and DSC2 showed increased and decreased expression alterations, respectively, in various epithelial cancers like head and neck^19^. Besides, enhanced tumor progression and reduced patient survival were observed on the expression reduction of gene DSG2^19^, while poor prognosis on the increased expression of DSC2^19^. Moreover, tumor development was observed via ectopic expression of DSG2 in mouse skin^20^. Also, gene expression analysis of PKP2, another desmosomal component, showed a significant correlation between its decreased expression and highly invasive tumors in pancreatic tissues^19^. Collectively, the desmosome down-regulation might contribute to malignant progression in tumors. Meanwhile in combination with our results, the module might also be related to inflammation progress. We therefore reasoned that, inflammation and cancer might employ certain common functional modules to facilitate their actions of expanding, where inflammation and cancer are tightly linked in local microenvironment.

Additionally, we also examined those overlapping edges in detail shared by both inflammation and cancer functional modules (Fig. 1). For example, common interaction IRS1 and JAK2 has been validated. As reported, insulin or IGF-1 receptor could active JAKs including JAK1 and JAK2, further directly phosphorylate IRS1 and IRS2 through the interactions between JAKs and IRSs^21^. Also, IRS1 was suggested to be activated via PTK2-mediated JNK activation^22^, which played vital roles in cell adhesion and survival under both inflammation and cancer^23^.

### Inflammation contributing to cancer through significant crosstalk with cancer modules

In addition to those directly overlapping nodes and edges serving as versatile players underlying the link between inflammation and cancer, we also explored other alternative approaches that inflammation might contribute to cancer in esophagus. Significant crosstalk module pairs of inflammation and cancer were thus determined based on permutation test, via randomizing PPI network with degree distribution of nodes keeping unchanged 1000 times^24^,^25^. With a p value cutoff of 0.05, we identified 402 significant crosstalk pairs in total (Supplementary Table 4).

**Figure 2:**
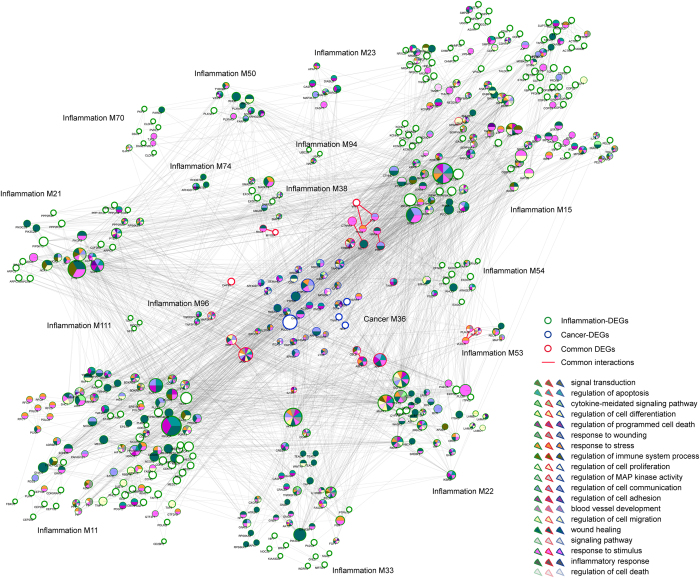
Inflammation contributes to cancer through significant module crosstalk. Module crosstalk was determined as significant, if the number of their interactions were significant more than expected. Functions based on GO analysis was conducted using Cytoscape plugin golorize. The border of nodes are colored as inflammation, cancer or common DEGs, with common interactions shown in red. Functions enriched by inflammation-, cancer- or common DEGs are also colored shown inside each node. Node size is shown according to its network degree. Module No. is also shown beside corresponding module.

Accordingly, we observed that some cancer-related module tended to connect with multiple inflammation-related modules (Fig. 2). As reported previously, inflammatory mediators and effectors are vital in the local micro-environment of tumors, which might present before malignant transformation of normal cells^26^. Local chronic inflammatory conditions affect all stages of tumor progression, especially the early onset of diverse diseases such as cancer^27^.

Based on functional analysis, we observed that DEGs from the central cancer module tended to significantly enrich in multiple disease related functions, such as signaling pathway, regulation of cell adhesion and regulation of programmed cell death and apoptosis. While, inflammation modules surrounded were enriched in other Gene ontology (GO) categories, for example regulation of cell proliferation, response to stimulus and wound healing. As indicated, one inflammation module (No. 11) and one cancer module (No. 36) not only shared common edges and DEGs, but also tightly connected via crosstalk interactions with part of module DEGs participating in the same or similar functions, for instance blood vessel development and regulation of immune system process. Overlapping gene JAK2 between the two modules, with a high degree indicated large impacts on the local subnetwork. Also, we observed that the gene participated in more functions than other DEGs from cancer or inflammation module. Previous studies reported that the aberrant expression of JAK2 was tightly correlated with the development of esophageal cancer^28^. As proofed, inhibition of JAK2 could block the inflammation and growth process of esophageal cancer *in vitro* through JAK/STAT3 pathway^28^, which thus indicated its pro-tumorigenic effects underlying the link between inflammation and cancer. We showed that those crosstalk interactions connecting to the gene, in both modules (inflammation module No. 11, and cancer module No. 36), might thus be perturbed by dysregulated expression of the gene. Accordingly, functional conjunctions between the two modules demonstrated an alternative way for cancer to communicate with inflammation, via both overlapping hub genes and part DEGs from different modules participating in the same or similar functions.

Another two inflammation modules were most involved in function of response to stimulus (module No. 15) and wound healing (module No. 21), which played pivotal roles in immune response to perturbed tissue homeostasis further contributing to the progression of cancer^29^. Other tightly connected functions like regulation of cell communication and regulation of cell death were also involved in the two functional modules. Besides, we also observed some DEGs have not significantly enriched in any GO functions. For example, DEG PIK3R2 in inflammation module (No. 15) with a relative bigger network degree has been confirmed to relate to several other cancers, like endometrial cancer^30^, breast cancer^31^, ovarian cancer^32^ and hepatocellular cancer^33^. Recently, in esophageal cancer, miR-126 was confirmed to inhibit the proliferation and migration *in vitro* cell line experiments^34^. Via PI3K/AKT signaling pathway which functions in angiogenesis, invasion, metastasis and survival, miR-126 probably directly regulated its direct target PIK3R2 to complete the connection between inflammation and cancer. We therefore reasoned that the gene might be a potential factor functioning in the underlying link between inflammation and cancer in esophagus. Additionally, other small inflammation modules (like No. 74 and No. 54) not only participated in their own specific functions (for example, regulation of cell adhesion and blood vessel development, respectively) but also functioned together with the central cancer module to attend some common functions (for example wound healing). Nevertheless, due to the lack of current knowledge on the module, further efforts and energy are needed to explore its relationship to cancer.

### Cancer relates to inflammation through significant crosstalk with inflammation modules

**Figure 3:**
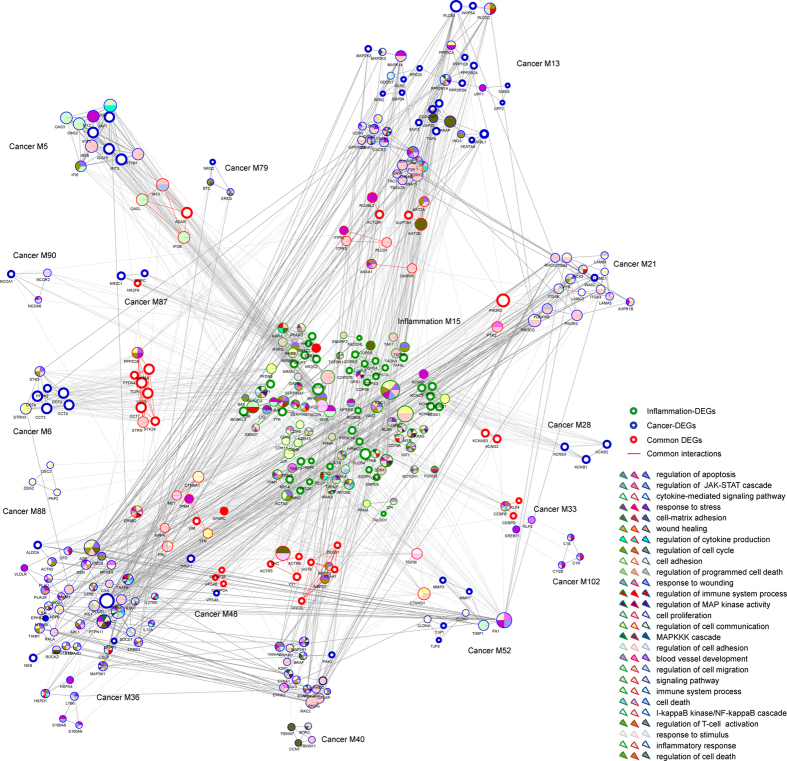
Cancer communicates with inflammation through significant crosstalk interactions. The central inflammation module is surrounded by several cancer modules, with common interactions shown in red. The border of nodes are colored as inflammation, cancer or common DEGs, with node size corresponding to their network degree. Functions enriched by inflammation-, cancer- or common DEGs are also colored shown inside each node. Module No. is listed beside corresponding module.

Conversely, in certain types of tumors, malignant changes induce an inflammatory microenvironment, which further promotes angiogenesis and metastasis in turn^35^. In established or late-stage cancers, accumulating evidence has suggested that local immune response and inflammatory mediators, though constituting a minor part, still played vital roles in progression of tumors and survival of patients with cancer. Accordingly, we observed a significant crosstalk subnetwork constituting an inflammation module and multiple cancer modules (Fig. 3).

We showed that DEGs from the central inflammation module were significantly enriched in multiple cancer-related functions, like cell proliferation, cell adhesion and regulation of MAP kinase activity, in spite of their inflammatory functions like immune system process. Those surrounding cancer modules not only had their unique functions like regulation of cell death, but also certain common functions with the inflammation module like regulation of cell communication and cell proliferation. Those common functions between the inflammation module and those cancer modules might thus be employed for established cancers to communicate with local inflammation and further fasten their metastasis and malignant transformation. As demonstrated, overlapping DEGs between one inflammation module (No. 15) and one cancer module (No. 6) formed a small tightly connected subnetwork, which significantly enriched in regulation of cell cycle, cell proliferation and signaling pathway. Other overlapping DEGs, were also partly involved in certain functions. For example, gene SPARC was reported to regulate inflammation during lung damage^36^ and promote metastatic dissemination of malignant cells in diverse human cancers, like melanoma, breast cancer and glioma^37^.

Additionally, we examined functions involved in other cancer modules, like module No. 36 and module No. 13. We observed that part DEGs from one cancer module (No. 36) participated in immune-related pathways, like cytokine-mediated signaling pathway, and regulation of T-cell activation. As previously reported, those immune responses were tightly associated with inflammatory stimuli^35^, which further contributed to the progression of cancer in turn. The other cancer module (No. 13) showed involvement in several other pathways, like cell proliferation and signaling pathway. Collectively, we demonstrated that, those cancer modules, though not fully understood in functions currently, might relate to inflammation via module genes participating in immune-related pathways.

### Transcriptional and post-transcriptional regulations involving in bridging inflammation and cancer in esophagus

With the development of computational predictors for studying the genomics^38^,^39^, individual transcription factors and microRNAs have been studied in linking inflammation and cancer^40^,^41^,^42^, for example NF-kappaB^43^ and microRNA-155^44^, however few research has focused on their actions on regulating significantly crosstalk modules. To explore those regulators, we applied pivot analysis on currently curated transcriptional and post-transcriptional regulations. According to threshold on both the number of regulatory interactions and significant p values, we identified 381 pivot microRNAs and 112 pivot TFs in total.

**Figure 4:**
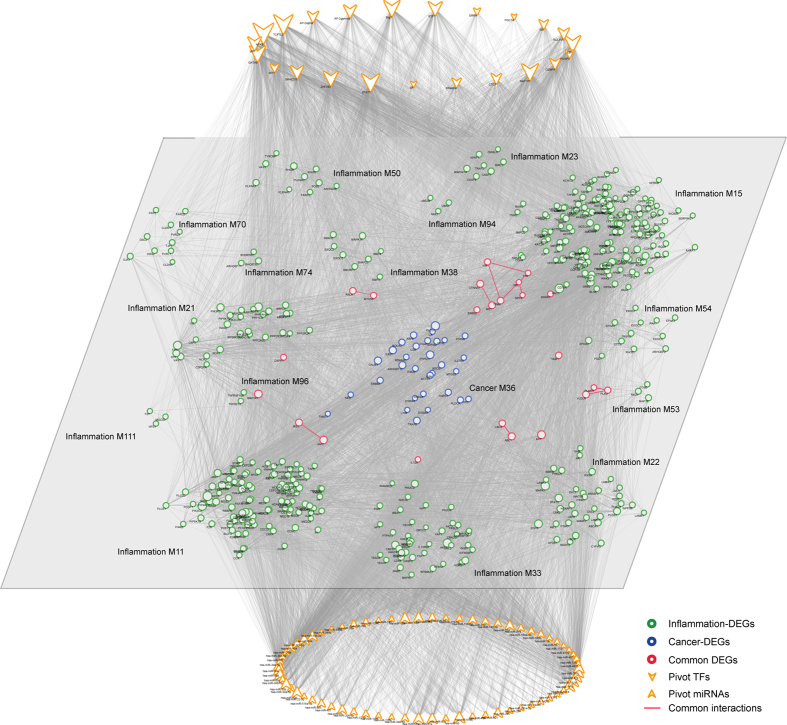
Inflammation contributes to cancer bridged by pivot TFs and miRNAs. Pivot regulators were identified based on both the number of their interactions within the module pair, and the significant enrichment of their regulated genes within per module. Pivot TFs are shown in the upper level of the network, with pivot miRNAs in the lower level. Module subnetwork comprises a central cancer module and several inflammation modules, with module No. beside them. The border of network nodes are colored as inflammation, cancer or common DEGs, with common interactions shown in red. Node size corresponds to its network degree.

**Figure 5:**
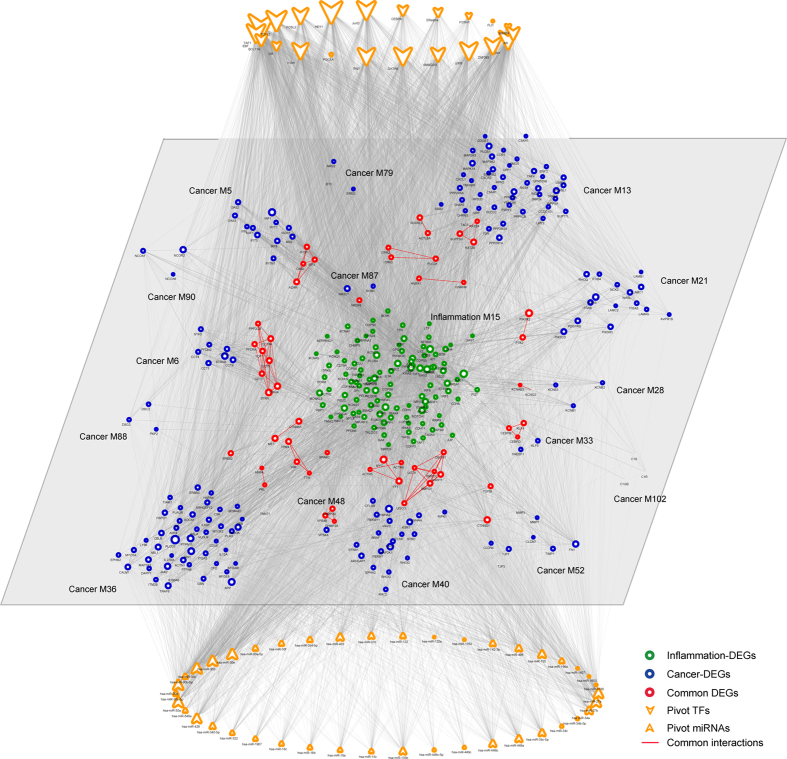
Cancer communicates with inflammation manipulated by pivot TFs and miRNAs. Pivot TFs are shown in the upper level of the network, with pivot miRNAs in the lower level. Module subnetwork comprises a central inflammation module and several surrounded cancer modules, with module No. marked beside them. The border of network nodes are colored as inflammation, cancer or common DEGs, with common interactions shown in red. Node size corresponds to its network degree.

According to the crosstalk module subnetworks described above, we extracted 23 pivot TFs and 69 pivot miRNAs that significantly regulated the subnetwork containing one central inflammation module and multiple cancer modules (Fig. 4). Similarly, we retrieved 22 pivot TFs and 42 pivot miRNAs for the subnetwork including one central cancer module and multiple inflammation modules (Fig. 5). According to network analysis on both module subnetworks, we observed that pivot regulators of TFs and miRNAs tended to target DEGs from both kinds of modules. Also those DEGs were mostly genes showing significant crosstalk interactions with other modules. Pivot TF BRG1 significantly regulated one cancer module (No. 36), and five inflammation modules (No. 11, No. 15, No. 21, No. 22, and No. 50) and ERalpha regulated cancer module (No. 36), and three inflammation modules (No. 15, No. 22, and No. 23) (Fig. 4). According to functional analysis, those modules were functionally involved in GO categories, such as apoptosis, cell death, proliferation and development. As proofed previously, BRG1 served as a tumor suppressor tightly connected to the oncogene MYC in primary tumors^45^, which promoted cell differentiation^45^ and transcription of pro-inflammatory genes^46^. The other TF ERalpha regulated cell growth and apoptosis in esophageal cancer^47^, which also showed a high consistency with functions enriched by its regulated modules. Moreover, we examined pivot miRNA miR-181 family (miR-181b, miR-181c and miR-181d), miR-27b and miR-200c, which significantly connected to the module subnetwork (Fig. 4). MiR-181b was identified as important in distinguishing low- from high-grade dysplasia in esophagus^48^, which could also regulate inflammation responses in immune cells^49^ and targeted by STAT3 as part of epigenetic switch linking inflammation and cancer^50^. Together with miR-27b, they were dysregulated showing up- and down-regulation respectively, between high-grade dysplasia and esophageal cancer^48^. MiR-200c was key regulators of epithelial to mesenchymal transition, which was also known to modulate apoptosis, cell proliferation and migration via targeting corresponding genes upon malignant progression of inflammatory esophagus^51^. Collectively, we reasoned that those pivot regulators, both TFs and miRNAs together with their significantly regulated crosstalk module subnetwork played vital roles in bridging inflammation and cancer in esophagus.

Similarly, we explored those pivot regulators with high degrees in the other module subnetwork (Fig. 5). TF BRG1, TF CEBPA, miRNA miR-27b and miRNA miR-30 family members recurred with relatively high degrees. Pivot TF C/EBP complex containing both protein alpha and beta, which was suggested to facilitate epithelial transformation via inducing COX-2 expression in gastric cancer^52^. Moreover, the beta protein was observed to help maintain local inflammatory environment in gastrointestinal tract, which therefore promotes malignant progression^53^. Pivot miRNA miR-30 family significantly regulated most modules in the subnetwork. As reported previously, miR-30c could regulate NF-kappa B negatively underlying both inflammation and cancer^54^. In lung cancer, down-regulation of both miR-30b and miR-30c was demonstrated to inhibit cell proliferation^55^.

### Case study: significant crosstalk module pair with most common DEGs

**Figure 6:**
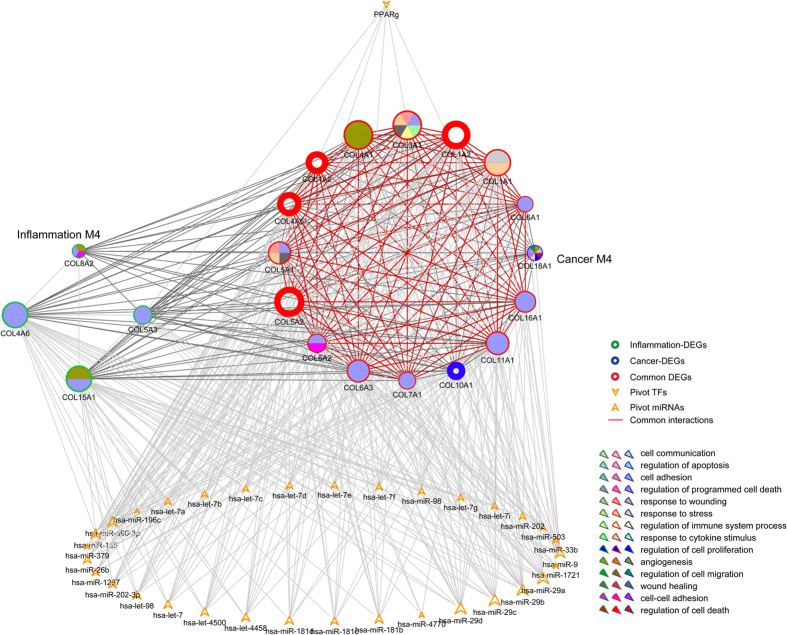
Module subnetwork showing both significant overlapping and crosstalk interactions. Module subnetwork contains one inflammation and one cancer module, with significant enriched functions colored inside each node. Pivot TFs and miRNAs were also extracted for the module subnetwork. The border of network nodes are colored as inflammation, cancer or common DEGs, with common interactions shown in red.

Based on all the crosstalk module pairs we identified, most pairs shared common DEGs ranging from 1 to 17. Interestingly, we observed that one inflammation module (No. 4) and one cancer module (No. 4) not only significantly shared common DEGs (comprise 87.5% of the cancer module), but also were tightly connected to each other with significant crosstalk interactions. Together, we extracted pivot TFs and miRNAs that significantly regulated the module subnetwork (Fig. 6).

Basically, as major components of fibrillary collagens, aberrations of gene COL1, COL3, COL4 and COL5 could cause a wide range of diseases in diverse human tissues, such as bone and blood^56^. Also based on functional analysis, we showed that those overlapping genes, shared by both inflammation and cancer module were responsible for angiogenesis and cell adhesion. As reported previously, aberrant alterations of cell adhesion were involved in almost every step of tumor progression, from the detachment of tumor cells at the primary sites to the formation of the secondary lesions^57^. Together with angiogenesis, we reasoned that these two functions might be employed by inflammation to contribute to cancer progression. Besides, we also examined those pivot regulators involved in regulating the module subnetwork. As demonstrated, most of the pivots (one pivot TF and 33 pivot miRNAs) tended to regulate those common genes with common and significant crosstalk interactions, which are consistent with our findings. Via examining the degree distribution of those pivot miRNAs, we found out that miR-29 family, miR-26b and miR-186 were among those top-ranked miRNAs. In mouse models, miR-29 family has been validated to regulate both innate and adaptive immune responses^58^. Additionally, the family was also correlated with diverse human malignances, such as lung cancer^59^ and hepatocellular cancer^60^. Recently, an aberrant down-regulation of miR-29a was observed in head-and-neck cancers^61^, which therefore indicated a potential role for the family in linking inflammation and cancer in esophagus. Besides, we reviewed that miR-186 and miR-26b were involved in cell cycle^62^ and cell growth regulation^63^, respectively. The only pivot TF PPARg was suggested to play a vital role in inflammation^64^. Even though, further evidence was needed for validating their roles in regulating the module subnetwork, we showed that they might function through both cell cycle regulation and cell adhesion to complete the underlying link in esophagus.

## Discussion

Even though the close link between inflammation and cancer has been generally accepted in most human cancers, large-scale explorations based on functional inflammation and cancer modules in esophagus are still lacking. We integrated multiple resources, such as gene expression alterations, protein-protein interaction network, transcriptional and post-transcriptional regulation data, to study the underlying link between inflammation and cancer in esophagus. In combination with crosstalk and pivot analysis, we showed that our functional module-based approach could not only include mechanisms that were already validated previously, but also provide a rich resource for potential candidate genes, interactions, pivot TFs and miRNAs that might play pivotal functions underlying the link in esophagus.

In addition to those important common DEGs and interactions shared by both inflammation and cancer modules, we also identified significant crosstalk module pairs of inflammation and cancer and then analyzed those module pairs at a network level. As indicated by those functional module pairs, we observed two different patterns, one of cancer module connected with multiple inflammation modules, and the other of inflammation module connected with multiple cancer modules. Functional analysis showed that, those common DEGs between module pairs played important roles underlying the tight connection between inflammation and cancer in esophagus. Based on manually annotations, we reasoned that common DEGs like PKP2, DSG2 and DSC2, and common edges like interaction between IRS1 and JAK2 as potential candidates for future biological validation. Together, we also generated pivot regulators of TFs and miRNAs, which served as important transcriptional and post-transcriptional regulators regulating the module subnetworks. Among these pivots, we showed that TF BRG1 and CEBPA and miRNA miR-27b and miR-30 family members recurred with relatively high degrees between the two different patterns, which thus turned out to be promising candidates for further confirmation. Besides, we also observed an interesting module pair, which showed significant crosstalk and meanwhile included the most common DEGs. Functional analysis suggested that they might act with pivot regulator through regulating cell cycle regulation and cell adhesion to complete the underlying link in esophagus.

Additionally, we also computed differentially expressed genes between disease condition of inflammation and cancer via applying the same algorithm and parameters, which resulted in 5267 inflammation-cancer DEGs (InCa-DEGs) in total. In comparison with those DEGs identified from inflammation-normal and cancer-normal, we observed 2020 and 1848 overlapped genes, respectively. Most InCa-DEGs were differentially expressed under only one disease condition in comparison with normal status. We missed most common DEGs, when overlapping common DEGs with InCa-DEGs. Some are supported as important in linking inflammation and cancer, for example PKP2, DSG2 and DSC2. Although, we focused on the crosstalk between functional module pairs, we hopefully consider it as an efficient complementary to our integrated approach that analyzes the network properties of those InCa-DEGs after mapping to the human PPI network in our future work.

Collectively, we applied a module-based methodology to explore the link between inflammation and cancer. In our results, we not only demonstrated the recurrence of those known factors, but also provided a rich resource of potential candidates for future experimental validations.

## Materials and Methods

### Data resources

Gene expression datasets were collected from the Gene Expression Omnibus^9^ (GEO) (http://www.ncbi.nlm.nih.gov/geo/) at the National Center for Biotechnology Information (NCBI).We downloaded CEL files of normal, esophagitis and esophageal cancer (GSE26886, GSE39491, GSE36223, GSE29001 and GSE20347), all of which were assessed using Affymetrix Human Genome U133 Plus 2.0 Array, together with an approximately equal number of normal and disease samples (see Table 1). We further applied least variant set (LVS) method for normalized expression profiling generation^65^,^66^,^67^,^68^.

Regulatory interactions containing transcriptional regulations and post-transcriptional regulations were downloaded from database ChIPBase^69^, TargetScan^70^ and miRanda^71^. From ChIPBase, 120 transcription factor (TF) and 323951 target interactions (TF-target) were collected. Meanwhile, we downloaded combined microRNA and target interactions from database of TargetScan, miRanda and RAID^72^, which resulted in 445 microRNAs together with 454176 interactions.

### Identifying differentially expressed genes (DEGs)

R package SAM was employed to compute DEGs between disease and normal samples in each dataset^10^. For per comparison between inflammation and normal, or cancer and normal, we determined inflammation- or cancer-DEGs with a 1% false discovery rate (FDR), respectively. Finally, we integrated the DEGs from all datasets to get high-confidence DEGs according to the following criteria: 1) the DEG was identified as differentially expressed in at least two datasets; 2) the dysregulated direction of the DEG, i.e. up- or down-regulation, was consistent.

### Generating inflammation- and cancer-related functional modules

The database STRING (Search Tool for the Retrieval of Interacting Genes/Proteins)is dedicated to protein-protein interactions (PPI), which provides the most comprehensive view on PPIs currently available, and thus acts as a meta-database for extensive PPI analysis^18^. We extracted a PPI network, containing 9061 proteins and 69400 high-confidence interactions with a score cutoff of 0.9. And then inflammation- and cancer-DEGs were mapped onto the PPI network. The maximal connected components (MCC) were subsequently obtained.

Based on the MCCs generated above, we identified inflammation- and cancer- related functional modules, via applying a well-developed MCODE method^17^ with default parameters.

### Determining significant crosstalk module pairs of inflammation and cancer

Based on the assumption that the crosstalk between inflammation- and cancer-related module is significant when the number of their interactions is significantly more than random distribution, we constructed 1000 random PPI network, with the degree distribution of nodes in the original network remaining unchanged^24^,^25^. For per pair of inflammation- and cancer-related module, we compared the real number of interactions between the module pair and the random distribution extracted from 1000 random PPI networks. The p value was computed as follows:
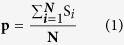
where S_i_ = 1 represents that the number of random interactions between the two modules was more than the real one, otherwise 0. The crosstalk between per module pair was defined as significant with a p value less than 0.05.

### Identifying pivot regulators

Pivot regulators were defined as regulators, including both microRNAs and TFs, significantly regulated both inflammation- and cancer-related module. We required that, the number of regulations between each regulator and per module of the pair was more than 2, and meanwhile a significant proportion of its targets enriched in each module determined by hypergeometric test with a p value was less than 0.05^73^.

### Gene ontology enrichment analysis

Significant gene ontology (GO) categories (‘biological processes’) were enriched using Cytoscape plugin golorize^74^, with a FDR less than 0.05.

## Additional Information

**How to cite this article**: Liu, N. *et al.* A functional module-based exploration between inflammation and cancer in esophagus. *Sci. Rep.* **5**, 15340; doi: 10.1038/srep15340 (2015).

## Supplementary Material

Supplementary Information

## Figures and Tables

**Table 1:** The microarray datasets summary.

**Accession id**	**Normal**	**Esophagitis**	**Esophageal cancer(EC)**
GSE26886	19*	20	30
GSE39491	40	40	–
GSE36223	23	23	–
GSE20347	17	–	17
GSE29001	12	–	21

*means the count of samples in dataset; - means no samples under that condition.
