# Supplementary material for: A functional module-based exploration between inflammation and cancer in esophagus
Source: Sci Rep. 2015 Oct 22;5:15340. doi: 10.1038/srep15340 (PMC4614801; doi:10.1038/srep15340)
Supplement: Supplementary Information [file srep15340-s1.doc]

**A functional module-based exploration between inflammation and cancer in esophagus**

Nannan Liu^3,†^, Chunhua Li^1,†^, Yan Huang^1,†^, Ying Yi^1^, Wanlan Bo^3^, Chunmiao Li^3^, Yue Li^3^, Yongfei Hu^1^, Kongning Li^1^, Hong Wang^2,*^, Liwei Zhuang^3,*^, Huihui Fan^1,*^, Dong Wang^1,*^

^1^College of Bioinformatics Science and Technology, Harbin Medical University, Harbin, Heilongjiang 150086, China

^2^Department of Network Information Center, Harbin Medical University, Harbin, Heilongjiang 150086, China

^3^The Fourth Affiliated Hospital, Harbin Medical University, Harbin, Heilongjiang 150001, China

†These authors contributed equally to this work.

*Corresponding to: Dong Wang, Email: wangdong@ems.hrbmu.edu.cn; Huihui Fan, Email: fanhuihui@hrbmu.edu.cn; Liwei Zhuang, Email:zhuangliweiyd@126.com; Hong Wang, Email: wanghong@ems.hrbmu.edu.cn.

| Supplementary Table 1.The validated cancer genes. | |
| --- | --- |
| Gene list | Source of Database |
| TP53 | CTD_human, GAD, BeFree |
| PTGS2 | CTD_human, BeFree |
| CDKN2A | CTD_human, BeFree |
| PIK3CA | CTD_human, BeFree |
| NELL1 | CTD_human, BeFree |
| SMAD4 | CTD_human, BeFree |
| ARID1A | CTD_human |
| DOCK2 | CTD_human |
| ELMO1 | CTD_human |
| SPG20 | CTD_human |
| TLR4 | CTD_human |
| TGFBR2 | UNIPROT, BeFree |
| CDKN2A | CTD_human, RGD, LHGDN, BeFree |
| PTGS2 | CTD_human, RGD, LHGDN, BeFree |
| SOD2 | CTD_human, RGD, LHGDN |
| CXCL2 | CTD_human, RGD |
| PTGS1 | CTD_human, RGD |
| TP53 | CTD_human, LHGDN, BeFree |
| ALDH2 | CTD_human, GAD, LHGDN, BeFree |
| ADH1B | CTD_human, GAD, LHGDN, BeFree |
| CCND1 | CTD_human, LHGDN, BeFree |
| EGFR | CTD_human, LHGDN, BeFree |
| BCL2 | CTD_human, LHGDN, BeFree |
| BAX | CTD_human, LHGDN, BeFree |
| RUNX3 | CTD_human, LHGDN, BeFree |
| SERPINB3 | CTD_human, LHGDN |
| XIAP | CTD_human, LHGDN |
| ERBB2 | CTD_human, BeFree |
| ABCB1 | CTD_human, BeFree |
| DEC1 | CTD_human, BeFree |
| ABL1 | CTD_human, BeFree |
| ALOX15 | CTD_human, BeFree |
| CASP8 | CTD_human, BeFree |
| CSF3 | CTD_human, BeFree |
| LGALS3 | CTD_human, BeFree |
| SFN | CTD_human, BeFree |
| XRCC3 | CTD_human, BeFree |
| ACTB | CTD_human |
| ADH7 | CTD_human |
| AKAP13 | CTD_human |
| AQP3 | CTD_human |
| BBC3 | CTD_human |
| BCL2A1 | CTD_human |
| CDH13 | CTD_human |
| CRYAB | CTD_human |
| CYP19A1 | CTD_human |
| DLEC1 | CTD_human |
| ENO1 | CTD_human |
| GAPDH | CTD_human |
| GHRL | CTD_human |
| GNG7 | CTD_human |
| KDR | CTD_human |
| LZTS1 | CTD_human |
| MAP3K3 | CTD_human |
| MARCH1 | CTD_human |
| MET | CTD_human |
| MIR145 | CTD_human |
| MIR200C | CTD_human |
| MIR21 | CTD_human |
| MLH3 | CTD_human |
| MMP14 | CTD_human |
| MT1G | CTD_human |
| NOS2 | CTD_human |
| NOS3 | CTD_human |
| PHB | CTD_human |
| PRDX2 | CTD_human |
| RNF6 | CTD_human |
| RPRM | CTD_human |
| SLC30A7 | CTD_human |
| SNAI2 | CTD_human |
| SST | CTD_human |
| TGFBR2 | CTD_human |
| TGM2 | CTD_human |
| TNFRSF10A | CTD_human |
| TPM1 | CTD_human |
| TPM4 | CTD_human |
| TUFM | CTD_human |
| UCHL1 | CTD_human |
| WIF1 | CTD_human |
| WWOX | CTD_human |
| ALDH2 | CTD_human, BeFree |
| EGFR | CTD_human, BeFree |
| PTGS2 | CTD_human, BeFree |
| ADH1B | CTD_human, BeFree |
| XRCC1 | CTD_human, BeFree |
| PLCE1 | CTD_human, BeFree |
| CALR | CTD_human, BeFree |
| CDKN1A | CTD_human, BeFree |
| CYP2A6 | CTD_human, BeFree |
| GSTT1 | CTD_human, BeFree |
| HIF1A | CTD_human, BeFree |
| SOX2 | CTD_human, BeFree |
| SFN | CTD_human, BeFree |
| ABCC2 | CTD_human, BeFree |
| ANXA1 | CTD_human, BeFree |
| CFL1 | CTD_human, BeFree |
| CRABP1 | CTD_human, BeFree |
| EP300 | CTD_human, BeFree |
| HMGB1 | CTD_human, BeFree |
| KRT14 | CTD_human, BeFree |
| KRT19 | CTD_human, BeFree |
| SLC39A6 | CTD_human, BeFree |
| SLC52A3 | CTD_human, BeFree |
| TNFAIP8 | CTD_human, BeFree |
| TPM1 | CTD_human, BeFree |
| TYMP | CTD_human, BeFree |
| ADCYAP1 | CTD_human |
| B2M | CTD_human |
| BLVRB | CTD_human |
| BUB1B | CTD_human |
| CA1 | CTD_human |
| CA2 | CTD_human |
| CAPZA1 | CTD_human |
| CLIC1 | CTD_human |
| CSTB | CTD_human |
| FKBP1A | CTD_human |
| GDI2 | CTD_human |
| GLO1 | CTD_human |
| GSTO1 | CTD_human |
| HIST1H2BM | CTD_human |
| HSPA5 | CTD_human |
| KRT13 | CTD_human |
| KRT17 | CTD_human |
| MAD2L1 | CTD_human |
| PFN1 | CTD_human |
| PRDX1 | CTD_human |
| PSME1 | CTD_human |
| PSME2 | CTD_human |
| SERPINB3 | CTD_human |
| SOD2 | CTD_human |
| TAGLN | CTD_human |
| TPI1 | CTD_human |
| TPM2 | CTD_human |
| TXNDC17 | CTD_human |
| ALDH2 | OMIM |
| ASCC1, p50 | OMIM |
| CTHRC1 | OMIM |
| MSR1, SCARA1, SRA | OMIM |
| DLEC1, DLC1 | OMIM |
| TGFBR2, HNPCC6, AAT3, MFS2, LDS1B, LDS2B | OMIM |
| RNF6 | OMIM |
| LZTS1, F37, FEZ1 | OMIM |
| WWOX, FOR | OMIM |

| Supplementary Table 2. Statistic information about inflammation-related functional modules. | | | |
| --- | --- | --- | --- |
| **No. of Module** | **Score (Density*#Nodes) from MCODE** | **Count of Nodes** | **Count of Edges** |
| 1 | 24.981 | 53 | 1324 |
| 2 | 15.343 | 35 | 537 |
| 3 | 9.217 | 23 | 212 |
| 4 | 8.333 | 18 | 150 |
| 5 | 7.571 | 21 | 159 |
| 6 | 7.062 | 16 | 113 |
| 7 | 6 | 14 | 84 |
| 8 | 5.714 | 14 | 80 |
| 9 | 5.5 | 12 | 66 |
| 10 | 5.2 | 15 | 78 |
| 11 | 4.305 | 105 | 452 |
| 12 | 4.057 | 35 | 142 |
| 13 | 3.8 | 10 | 38 |
| 14 | 3.6 | 10 | 36 |
| 15 | 3.364 | 154 | 518 |
| 16 | 3.125 | 8 | 25 |
| 17 | 2.941 | 17 | 50 |
| 18 | 2.857 | 7 | 20 |
| 19 | 2.455 | 11 | 27 |
| 20 | 2.375 | 8 | 19 |
| 21 | 2.25 | 28 | 63 |
| 22 | 2.056 | 36 | 74 |
| 23 | 2 | 7 | 14 |
| 24 | 2 | 13 | 26 |
| 25 | 2 | 5 | 10 |
| 26 | 2 | 5 | 10 |
| 27 | 2 | 5 | 10 |
| 28 | 2 | 5 | 10 |
| 29 | 2 | 5 | 10 |
| 30 | 2 | 5 | 10 |
| 31 | 1.909 | 11 | 21 |
| 32 | 1.857 | 7 | 13 |
| 33 | 1.805 | 41 | 74 |
| 34 | 1.8 | 5 | 9 |
| 35 | 1.667 | 12 | 20 |
| 36 | 1.667 | 9 | 15 |
| 37 | 1.6 | 5 | 8 |
| 38 | 1.6 | 10 | 16 |
| 39 | 1.6 | 5 | 8 |
| 40 | 1.556 | 18 | 28 |
| 41 | 1.5 | 4 | 6 |
| 42 | 1.5 | 4 | 6 |
| 43 | 1.5 | 4 | 6 |
| 44 | 1.5 | 4 | 6 |
| 45 | 1.5 | 4 | 6 |
| 46 | 1.5 | 4 | 6 |
| 47 | 1.5 | 4 | 6 |
| 48 | 1.474 | 19 | 28 |
| 49 | 1.429 | 7 | 10 |
| 50 | 1.4 | 10 | 14 |
| 51 | 1.4 | 5 | 7 |
| 52 | 1.375 | 8 | 11 |
| 53 | 1.333 | 6 | 8 |
| 54 | 1.308 | 13 | 17 |
| 55 | 1.286 | 7 | 9 |
| 56 | 1.25 | 4 | 5 |
| 57 | 1.25 | 4 | 5 |
| 58 | 1.25 | 4 | 5 |
| 59 | 1.25 | 4 | 5 |
| 60 | 1.25 | 4 | 5 |
| 61 | 1.25 | 4 | 5 |
| 62 | 1.25 | 4 | 5 |
| 63 | 1.25 | 4 | 5 |
| 64 | 1.25 | 4 | 5 |
| 65 | 1.25 | 4 | 5 |
| 66 | 1.25 | 4 | 5 |
| 67 | 1.25 | 4 | 5 |
| 68 | 1.25 | 4 | 5 |
| 69 | 1.222 | 9 | 11 |
| 70 | 1.2 | 10 | 12 |
| 71 | 1.2 | 5 | 6 |
| 72 | 1.167 | 6 | 7 |
| 73 | 1 | 3 | 3 |
| 74 | 1 | 3 | 3 |
| 75 | 1 | 3 | 3 |
| 76 | 1 | 3 | 3 |
| 77 | 1 | 3 | 3 |
| 78 | 1 | 3 | 3 |
| 79 | 1 | 3 | 3 |
| 80 | 1 | 3 | 3 |
| 81 | 1 | 3 | 3 |
| 82 | 1 | 3 | 3 |
| 83 | 1 | 3 | 3 |
| 84 | 1 | 3 | 3 |
| 85 | 1 | 3 | 3 |
| 86 | 1 | 3 | 3 |
| 87 | 1 | 3 | 3 |
| 88 | 1 | 3 | 3 |
| 89 | 1 | 3 | 3 |
| 90 | 1 | 3 | 3 |
| 91 | 1 | 3 | 3 |
| 92 | 1 | 3 | 3 |
| 93 | 1 | 3 | 3 |
| 94 | 1 | 3 | 3 |
| 95 | 1 | 3 | 3 |
| 96 | 1 | 3 | 3 |
| 97 | 1 | 3 | 3 |
| 98 | 1 | 3 | 3 |
| 99 | 1 | 3 | 3 |
| 100 | 1 | 3 | 3 |
| 101 | 1 | 3 | 3 |
| 102 | 1 | 3 | 3 |
| 103 | 1 | 3 | 3 |
| 104 | 1 | 3 | 3 |
| 105 | 1 | 3 | 3 |
| 106 | 1 | 3 | 3 |
| 107 | 1 | 3 | 3 |
| 108 | 1 | 3 | 3 |
| 109 | 1 | 3 | 3 |
| 110 | 1 | 3 | 3 |
| 111 | 1 | 3 | 3 |
| 112 | 1 | 3 | 3 |
| 113 | 1 | 3 | 3 |
| 114 | 1 | 4 | 4 |
| 115 | 1 | 3 | 3 |
| 116 | 1 | 4 | 4 |
| 117 | 1 | 4 | 4 |
| 118 | 1 | 4 | 4 |

| Supplementary Table 3. Statistics about cancer-related functional modules | | | |  |
| --- | --- | --- | --- | --- |
| **No. of Module** | **Score (Density*#Nodes) from MCODE** | **Count of Nodes** | **Count of Edges** | |
| 1 | 17.076 | 79 | 1349 | |
| 2 | 9 | 19 | 171 | |
| 3 | 7.96 | 25 | 199 | |
| 4 | 7.375 | 16 | 118 | |
| 5 | 6.867 | 15 | 103 | |
| 6 | 6.133 | 15 | 92 | |
| 7 | 6.12 | 25 | 153 | |
| 8 | 4.4 | 10 | 44 | |
| 9 | 3.885 | 26 | 101 | |
| 10 | 3.818 | 11 | 42 | |
| 11 | 3.5 | 8 | 28 | |
| 12 | 3.312 | 16 | 53 | |
| 13 | 3.17 | 53 | 168 | |
| 14 | 2.857 | 7 | 20 | |
| 15 | 2.571 | 7 | 18 | |
| 16 | 2.556 | 9 | 23 | |
| 17 | 2.5 | 6 | 15 | |
| 18 | 2.5 | 10 | 25 | |
| 19 | 2.467 | 15 | 37 | |
| 20 | 2.44 | 25 | 61 | |
| 21 | 2.353 | 17 | 40 | |
| 22 | 2.333 | 6 | 14 | |
| 23 | 2.167 | 6 | 13 | |
| 24 | 2.167 | 6 | 13 | |
| 25 | 2 | 8 | 16 | |
| 26 | 2 | 5 | 10 | |
| 27 | 2 | 5 | 10 | |
| 28 | 2 | 5 | 10 | |
| 29 | 2 | 5 | 10 | |
| 30 | 2 | 5 | 10 | |
| 31 | 2 | 5 | 10 | |
| 32 | 2 | 5 | 10 | |
| 33 | 2 | 5 | 10 | |
| 34 | 2 | 5 | 10 | |
| 35 | 2 | 6 | 12 | |
| 36 | 1.889 | 54 | 102 | |
| 37 | 1.875 | 16 | 30 | |
| 38 | 1.857 | 14 | 26 | |
| 39 | 1.8 | 5 | 9 | |
| 40 | 1.793 | 29 | 52 | |
| 41 | 1.7 | 10 | 17 | |
| 42 | 1.6 | 5 | 8 | |
| 43 | 1.545 | 11 | 17 | |
| 44 | 1.5 | 10 | 15 | |
| 45 | 1.5 | 4 | 6 | |
| 46 | 1.5 | 4 | 6 | |
| 47 | 1.5 | 4 | 6 | |
| 48 | 1.5 | 4 | 6 | |
| 49 | 1.5 | 4 | 6 | |
| 50 | 1.5 | 6 | 9 | |
| 51 | 1.5 | 6 | 9 | |
| 52 | 1.5 | 10 | 15 | |
| 53 | 1.5 | 4 | 6 | |
| 54 | 1.5 | 4 | 6 | |
| 55 | 1.444 | 18 | 26 | |
| 56 | 1.4 | 5 | 7 | |
| 57 | 1.25 | 4 | 5 | |
| 58 | 1.25 | 4 | 5 | |
| 59 | 1.25 | 4 | 5 | |
| 60 | 1.25 | 4 | 5 | |
| 61 | 1.25 | 4 | 5 | |
| 62 | 1.25 | 4 | 5 | |
| 63 | 1.25 | 4 | 5 | |
| 64 | 1.25 | 4 | 5 | |
| 65 | 1.25 | 4 | 5 | |
| 66 | 1.2 | 5 | 6 | |
| 67 | 1.2 | 5 | 6 | |
| 68 | 1 | 3 | 3 | |
| 69 | 1 | 3 | 3 | |
| 70 | 1 | 3 | 3 | |
| 71 | 1 | 3 | 3 | |
| 72 | 1 | 3 | 3 | |
| 73 | 1 | 3 | 3 | |
| 74 | 1 | 3 | 3 | |
| 75 | 1 | 3 | 3 | |
| 76 | 1 | 3 | 3 | |
| 77 | 1 | 3 | 3 | |
| 78 | 1 | 3 | 3 | |
| 79 | 1 | 3 | 3 | |
| 80 | 1 | 3 | 3 | |
| 81 | 1 | 3 | 3 | |
| 82 | 1 | 3 | 3 | |
| 83 | 1 | 3 | 3 | |
| 84 | 1 | 3 | 3 | |
| 85 | 1 | 3 | 3 | |
| 86 | 1 | 3 | 3 | |
| 87 | 1 | 3 | 3 | |
| 88 | 1 | 3 | 3 | |
| 89 | 1 | 3 | 3 | |
| 90 | 1 | 3 | 3 | |
| 91 | 1 | 3 | 3 | |
| 92 | 1 | 3 | 3 | |
| 93 | 1 | 3 | 3 | |
| 94 | 1 | 3 | 3 | |
| 95 | 1 | 3 | 3 | |
| 96 | 1 | 3 | 3 | |
| 97 | 1 | 3 | 3 | |
| 98 | 1 | 3 | 3 | |
| 99 | 1 | 3 | 3 | |
| 100 | 1 | 3 | 3 | |
| 101 | 1 | 3 | 3 | |
| 102 | 1 | 3 | 3 | |
| 103 | 1 | 3 | 3 | |
| 104 | 1 | 3 | 3 | |
| 105 | 1 | 3 | 3 | |
| 106 | 1 | 3 | 3 | |
| 107 | 1 | 3 | 3 | |
| 108 | 1 | 3 | 3 | |
| 109 | 1 | 3 | 3 | |
| 110 | 1 | 3 | 3 | |

| Supplementary Table 4. Statistics about significant crosstalk module pairs of inflammation and cancer. | | | |
| --- | --- | --- | --- |
| **No. of inflammation_module** | **No. of cancer_module** | **Count of interactions** | **p.value** |
| 1 | 10 | 482 | 0 |
| 1 | 13 | 177 | 0 |
| 1 | 30 | 6 | 0.017 |
| 1 | 31 | 4 | 0.036 |
| 2 | 2 | 28 | 0.035 |
| 2 | 7 | 500 | 0 |
| 2 | 44 | 171 | 0 |
| 2 | 46 | 40 | 0 |
| 2 | 48 | 8 | 0 |
| 2 | 94 | 1 | 0.047 |
| 2 | 109 | 2 | 0.018 |
| 3 | 1 | 383 | 0 |
| 3 | 3 | 429 | 0 |
| 3 | 19 | 125 | 0 |
| 3 | 20 | 36 | 0 |
| 3 | 39 | 8 | 0 |
| 3 | 43 | 14 | 0 |
| 3 | 47 | 10 | 0 |
| 3 | 49 | 3 | 0.007 |
| 4 | 4 | 178 | 0 |
| 4 | 52 | 4 | 0.05 |
| 4 | 98 | 1 | 0.015 |
| 5 | 1 | 718 | 0 |
| 5 | 7 | 42 | 0.001 |
| 5 | 9 | 20 | 0.01 |
| 5 | 19 | 28 | 0 |
| 5 | 46 | 11 | 0 |
| 5 | 68 | 29 | 0 |
| 6 | 15 | 90 | 0 |
| 6 | 62 | 3 | 0 |
| 7 | 12 | 91 | 0 |
| 7 | 52 | 4 | 0.005 |
| 8 | 1 | 364 | 0 |
| 8 | 2 | 42 | 0 |
| 8 | 7 | 22 | 0.01 |
| 8 | 9 | 15 | 0 |
| 8 | 25 | 28 | 0 |
| 8 | 44 | 27 | 0 |
| 8 | 46 | 8 | 0 |
| 8 | 58 | 6 | 0 |
| 8 | 82 | 2 | 0 |
| 9 | 11 | 75 | 0 |
| 9 | 67 | 1 | 0.013 |
| 9 | 80 | 7 | 0 |
| 10 | 1 | 63 | 0 |
| 10 | 2 | 196 | 0 |
| 10 | 7 | 43 | 0 |
| 10 | 9 | 12 | 0.001 |
| 10 | 20 | 11 | 0.007 |
| 10 | 25 | 37 | 0 |
| 10 | 43 | 5 | 0.026 |
| 10 | 44 | 29 | 0 |
| 10 | 45 | 13 | 0 |
| 10 | 58 | 6 | 0 |
| 10 | 69 | 10 | 0 |
| 10 | 73 | 3 | 0 |
| 11 | 2 | 63 | 0.001 |
| 11 | 8 | 120 | 0 |
| 11 | 9 | 134 | 0 |
| 11 | 13 | 186 | 0 |
| 11 | 20 | 81 | 0 |
| 11 | 21 | 57 | 0 |
| 11 | 23 | 36 | 0 |
| 11 | 25 | 38 | 0 |
| 11 | 33 | 19 | 0 |
| 11 | 36 | 124 | 0.003 |
| 11 | 40 | 64 | 0 |
| 11 | 45 | 26 | 0 |
| 11 | 55 | 42 | 0.001 |
| 11 | 67 | 8 | 0 |
| 11 | 71 | 13 | 0 |
| 11 | 90 | 34 | 0 |
| 12 | 1 | 257 | 0 |
| 12 | 3 | 185 | 0 |
| 12 | 7 | 43 | 0 |
| 12 | 19 | 145 | 0 |
| 12 | 20 | 66 | 0 |
| 12 | 39 | 3 | 0.019 |
| 12 | 43 | 45 | 0 |
| 12 | 47 | 4 | 0 |
| 12 | 49 | 9 | 0 |
| 12 | 50 | 6 | 0.001 |
| 12 | 101 | 4 | 0 |
| 13 | 16 | 1 | 0.018 |
| 13 | 30 | 1 | 0.004 |
| 13 | 39 | 5 | 0 |
| 13 | 75 | 24 | 0 |
| 14 | 14 | 46 | 0 |
| 14 | 54 | 2 | 0 |
| 15 | 5 | 127 | 0 |
| 15 | 6 | 118 | 0 |
| 15 | 13 | 190 | 0 |
| 15 | 21 | 63 | 0 |
| 15 | 28 | 41 | 0 |
| 15 | 33 | 15 | 0.002 |
| 15 | 36 | 253 | 0 |
| 15 | 40 | 93 | 0 |
| 15 | 48 | 14 | 0 |
| 15 | 52 | 47 | 0 |
| 15 | 79 | 3 | 0.025 |
| 15 | 87 | 22 | 0 |
| 15 | 88 | 3 | 0 |
| 15 | 90 | 12 | 0.028 |
| 15 | 102 | 3 | 0.01 |
| 16 | 11 | 12 | 0 |
| 16 | 80 | 21 | 0 |
| 17 | 24 | 2 | 0.016 |
| 17 | 26 | 19 | 0 |
| 17 | 41 | 76 | 0 |
| 17 | 51 | 46 | 0 |
| 17 | 64 | 1 | 0.003 |
| 17 | 66 | 10 | 0 |
| 18 | 101 | 18 | 0 |
| 19 | 24 | 6 | 0 |
| 19 | 38 | 43 | 0 |
| 19 | 43 | 2 | 0.046 |
| 19 | 44 | 6 | 0 |
| 19 | 50 | 4 | 0 |
| 20 | 24 | 3 | 0 |
| 20 | 26 | 11 | 0 |
| 20 | 41 | 34 | 0 |
| 20 | 51 | 25 | 0 |
| 20 | 66 | 4 | 0 |
| 21 | 6 | 7 | 0.017 |
| 21 | 13 | 49 | 0 |
| 21 | 21 | 46 | 0 |
| 21 | 32 | 24 | 0 |
| 21 | 36 | 58 | 0 |
| 21 | 40 | 19 | 0 |
| 21 | 55 | 12 | 0 |
| 21 | 74 | 3 | 0.002 |
| 21 | 89 | 14 | 0 |
| 21 | 108 | 1 | 0.03 |
| 22 | 18 | 15 | 0 |
| 22 | 21 | 48 | 0 |
| 22 | 32 | 12 | 0 |
| 22 | 33 | 7 | 0 |
| 22 | 36 | 76 | 0 |
| 22 | 52 | 11 | 0.005 |
| 22 | 55 | 23 | 0 |
| 22 | 61 | 18 | 0 |
| 22 | 67 | 5 | 0 |
| 22 | 72 | 4 | 0.001 |
| 22 | 79 | 2 | 0.006 |
| 22 | 81 | 1 | 0.025 |
| 22 | 87 | 3 | 0.014 |
| 22 | 89 | 23 | 0 |
| 22 | 90 | 7 | 0 |
| 22 | 93 | 1 | 0.025 |
| 22 | 107 | 4 | 0 |
| 23 | 18 | 10 | 0 |
| 23 | 36 | 6 | 0.038 |
| 23 | 40 | 5 | 0.003 |
| 24 | 12 | 2 | 0.041 |
| 24 | 24 | 16 | 0 |
| 24 | 29 | 13 | 0 |
| 24 | 38 | 5 | 0 |
| 24 | 41 | 9 | 0 |
| 24 | 51 | 4 | 0 |
| 24 | 54 | 1 | 0.005 |
| 24 | 60 | 2 | 0 |
| 25 | 55 | 17 | 0 |
| 25 | 67 | 1 | 0 |
| 26 | 22 | 25 | 0 |
| 27 | 34 | 22 | 0 |
| 29 | 41 | 2 | 0.001 |
| 30 | 26 | 3 | 0 |
| 30 | 37 | 16 | 0 |
| 31 | 24 | 4 | 0 |
| 31 | 25 | 2 | 0.05 |
| 31 | 38 | 14 | 0 |
| 31 | 43 | 6 | 0 |
| 31 | 50 | 6 | 0 |
| 31 | 52 | 2 | 0.03 |
| 31 | 106 | 1 | 0.005 |
| 32 | 18 | 8 | 0 |
| 32 | 37 | 20 | 0 |
| 33 | 13 | 42 | 0.011 |
| 33 | 16 | 28 | 0 |
| 33 | 17 | 33 | 0 |
| 33 | 18 | 18 | 0 |
| 33 | 21 | 14 | 0.011 |
| 33 | 33 | 4 | 0.03 |
| 33 | 36 | 47 | 0 |
| 33 | 40 | 20 | 0.007 |
| 33 | 55 | 24 | 0 |
| 33 | 85 | 2 | 0.015 |
| 33 | 103 | 1 | 0.018 |
| 33 | 110 | 3 | 0.01 |
| 34 | 43 | 1 | 0.018 |
| 34 | 54 | 13 | 0 |
| 35 | 12 | 2 | 0.016 |
| 35 | 20 | 6 | 0 |
| 35 | 35 | 1 | 0.024 |
| 35 | 57 | 11 | 0 |
| 36 | 7 | 4 | 0.041 |
| 36 | 10 | 2 | 0.046 |
| 36 | 30 | 18 | 0 |
| 37 | 1 | 105 | 0 |
| 37 | 23 | 2 | 0.002 |
| 37 | 68 | 10 | 0 |
| 37 | 71 | 3 | 0 |
| 38 | 18 | 4 | 0.009 |
| 38 | 21 | 5 | 0.011 |
| 38 | 36 | 17 | 0.002 |
| 38 | 37 | 19 | 0 |
| 38 | 55 | 5 | 0.019 |
| 38 | 58 | 2 | 0.028 |
| 38 | 84 | 1 | 0.009 |
| 38 | 89 | 6 | 0 |
| 39 | 1 | 11 | 0.005 |
| 39 | 3 | 8 | 0 |
| 39 | 47 | 3 | 0 |
| 39 | 85 | 1 | 0 |
| 39 | 86 | 1 | 0 |
| 40 | 8 | 8 | 0 |
| 40 | 9 | 33 | 0 |
| 40 | 20 | 25 | 0 |
| 40 | 33 | 12 | 0 |
| 40 | 39 | 4 | 0 |
| 40 | 40 | 12 | 0.04 |
| 40 | 43 | 13 | 0 |
| 40 | 47 | 9 | 0 |
| 40 | 55 | 13 | 0 |
| 40 | 58 | 5 | 0.002 |
| 40 | 72 | 6 | 0 |
| 40 | 87 | 3 | 0.005 |
| 40 | 90 | 11 | 0 |
| 40 | 101 | 1 | 0.04 |
| 41 | 26 | 14 | 0 |
| 41 | 37 | 3 | 0 |
| 41 | 41 | 10 | 0 |
| 41 | 51 | 7 | 0 |
| 41 | 64 | 1 | 0 |
| 42 | 65 | 8 | 0 |
| 45 | 3 | 2 | 0.021 |
| 45 | 10 | 1 | 0.037 |
| 45 | 78 | 11 | 0 |
| 46 | 20 | 2 | 0.001 |
| 46 | 40 | 1 | 0.018 |
| 47 | 52 | 1 | 0.009 |
| 48 | 38 | 4 | 0 |
| 48 | 51 | 9 | 0 |
| 48 | 60 | 6 | 0 |
| 48 | 66 | 9 | 0 |
| 48 | 83 | 1 | 0.001 |
| 49 | 5 | 17 | 0 |
| 49 | 33 | 1 | 0.005 |
| 49 | 44 | 1 | 0.036 |
| 49 | 70 | 8 | 0 |
| 50 | 21 | 7 | 0 |
| 50 | 36 | 10 | 0.02 |
| 50 | 40 | 21 | 0 |
| 50 | 55 | 6 | 0.002 |
| 50 | 74 | 7 | 0 |
| 50 | 77 | 3 | 0 |
| 50 | 89 | 10 | 0 |
| 50 | 102 | 1 | 0 |
| 52 | 11 | 3 | 0 |
| 52 | 90 | 1 | 0.032 |
| 52 | 103 | 1 | 0 |
| 53 | 21 | 9 | 0 |
| 53 | 36 | 11 | 0 |
| 53 | 52 | 2 | 0.012 |
| 53 | 55 | 5 | 0.001 |
| 53 | 71 | 1 | 0.009 |
| 53 | 77 | 1 | 0 |
| 53 | 92 | 1 | 0 |
| 54 | 21 | 10 | 0 |
| 54 | 32 | 1 | 0.01 |
| 54 | 36 | 16 | 0 |
| 54 | 40 | 26 | 0 |
| 54 | 52 | 5 | 0 |
| 54 | 55 | 8 | 0 |
| 54 | 74 | 1 | 0.005 |
| 54 | 89 | 5 | 0 |
| 55 | 7 | 5 | 0.005 |
| 55 | 9 | 2 | 0.039 |
| 55 | 23 | 1 | 0.008 |
| 55 | 40 | 14 | 0 |
| 55 | 55 | 6 | 0 |
| 55 | 83 | 1 | 0 |
| 56 | 1 | 35 | 0 |
| 56 | 3 | 14 | 0 |
| 56 | 7 | 16 | 0 |
| 56 | 19 | 25 | 0 |
| 57 | 23 | 17 | 0 |
| 57 | 68 | 2 | 0.001 |
| 57 | 71 | 7 | 0 |
| 58 | 2 | 3 | 0.003 |
| 58 | 19 | 2 | 0.026 |
| 58 | 20 | 16 | 0 |
| 58 | 25 | 2 | 0.004 |
| 58 | 73 | 3 | 0 |
| 59 | 55 | 5 | 0 |
| 59 | 66 | 3 | 0 |
| 59 | 72 | 4 | 0 |
| 59 | 76 | 4 | 0 |
| 60 | 15 | 4 | 0 |
| 61 | 7 | 6 | 0 |
| 61 | 9 | 14 | 0 |
| 61 | 20 | 18 | 0 |
| 61 | 25 | 4 | 0 |
| 61 | 44 | 2 | 0.015 |
| 61 | 45 | 11 | 0 |
| 61 | 51 | 1 | 0.024 |
| 61 | 58 | 3 | 0 |
| 62 | 42 | 4 | 0 |
| 62 | 44 | 4 | 0 |
| 62 | 73 | 8 | 0 |
| 63 | 13 | 16 | 0 |
| 63 | 110 | 1 | 0.005 |
| 64 | 7 | 2 | 0.007 |
| 65 | 41 | 2 | 0.005 |
| 65 | 51 | 1 | 0.019 |
| 66 | 16 | 2 | 0 |
| 66 | 17 | 4 | 0 |
| 66 | 42 | 11 | 0 |
| 66 | 43 | 1 | 0.031 |
| 66 | 50 | 1 | 0.002 |
| 66 | 56 | 10 | 0 |
| 67 | 93 | 5 | 0 |
| 69 | 53 | 7 | 0 |
| 69 | 61 | 1 | 0.004 |
| 70 | 36 | 4 | 0.041 |
| 70 | 52 | 7 | 0 |
| 70 | 88 | 4 | 0 |
| 71 | 20 | 3 | 0 |
| 71 | 40 | 3 | 0 |
| 72 | 33 | 1 | 0.003 |
| 73 | 10 | 7 | 0 |
| 74 | 21 | 3 | 0 |
| 74 | 36 | 4 | 0.008 |
| 74 | 37 | 1 | 0.017 |
| 74 | 40 | 7 | 0 |
| 74 | 89 | 1 | 0.008 |
| 75 | 7 | 2 | 0.032 |
| 75 | 12 | 1 | 0.011 |
| 75 | 43 | 3 | 0 |
| 75 | 44 | 2 | 0 |
| 75 | 107 | 1 | 0 |
| 76 | 14 | 15 | 0 |
| 77 | 43 | 8 | 0 |
| 77 | 55 | 3 | 0.001 |
| 77 | 90 | 1 | 0.002 |
| 78 | 13 | 17 | 0 |
| 78 | 20 | 3 | 0 |
| 79 | 10 | 3 | 0 |
| 80 | 12 | 7 | 0 |
| 80 | 59 | 8 | 0 |
| 81 | 13 | 3 | 0.024 |
| 81 | 33 | 2 | 0 |
| 82 | 35 | 9 | 0 |
| 83 | 71 | 5 | 0 |
| 84 | 21 | 13 | 0 |
| 86 | 10 | 1 | 0.039 |
| 86 | 37 | 1 | 0.011 |
| 87 | 7 | 1 | 0.047 |
| 87 | 25 | 1 | 0.003 |
| 88 | 90 | 3 | 0 |
| 89 | 2 | 1 | 0.035 |
| 89 | 25 | 7 | 0 |
| 89 | 45 | 1 | 0 |
| 89 | 58 | 1 | 0.002 |
| 90 | 42 | 2 | 0 |
| 92 | 35 | 6 | 0 |
| 92 | 106 | 1 | 0.001 |
| 93 | 26 | 1 | 0.003 |
| 93 | 37 | 9 | 0 |
| 93 | 110 | 1 | 0.002 |
| 94 | 36 | 1 | 0.047 |
| 95 | 42 | 11 | 0 |
| 95 | 56 | 10 | 0 |
| 96 | 12 | 2 | 0.004 |
| 96 | 18 | 13 | 0 |
| 96 | 36 | 5 | 0.003 |
| 96 | 40 | 9 | 0 |
| 96 | 89 | 1 | 0.005 |
| 97 | 38 | 4 | 0 |
| 98 | 26 | 2 | 0 |
| 98 | 33 | 1 | 0 |
| 104 | 63 | 5 | 0 |
| 106 | 27 | 6 | 0 |
| 106 | 86 | 3 | 0 |
| 107 | 76 | 2 | 0 |
| 107 | 83 | 5 | 0 |
| 108 | 104 | 3 | 0 |
| 109 | 71 | 3 | 0 |
| 110 | 82 | 1 | 0 |
| 111 | 36 | 1 | 0.035 |
| 112 | 29 | 1 | 0 |
| 112 | 54 | 2 | 0 |
| 112 | 108 | 2 | 0 |
| 114 | 24 | 1 | 0 |
| 114 | 99 | 1 | 0 |
| 114 | 108 | 7 | 0 |
| 115 | 41 | 3 | 0 |
| 115 | 59 | 3 | 0 |
| 117 | 38 | 2 | 0 |
| 117 | 95 | 1 | 0 |
| 118 | 35 | 1 | 0.003 |
| 118 | 106 | 5 | 0 |

| Supplementary Table 5. Statistics about pivot regulators of inflammation- and cancer- related functional modules. | | | | | |
| --- | --- | --- | --- | --- | --- |
| **No. of inflammation_module** | **No. of cancer_module** | **Count of interactions** | **p.value** | **Count of crosstalk_module related TFs** | **Count of crosstalk_module related miRNAs** |
| 1 | 10 | 482 | 0 | 39 | - |
| 1 | 13 | 177 | 0 | 11 | - |
| 1 | 30 | 6 | 0.017 | 10 | - |
| 1 | 31 | 4 | 0.036 | 11 | - |
| 2 | 2 | 28 | 0.035 | 31 | - |
| 2 | 7 | 500 | 0 | 45 | - |
| 2 | 44 | 171 | 0 | 27 | - |
| 2 | 46 | 40 | 0 | 5 | - |
| 2 | 48 | 8 | 0 | 4 | - |
| 2 | 94 | 1 | 0.047 | - | - |
| 2 | 109 | 2 | 0.018 | 3 | - |
| 3 | 1 | 383 | 0 | 61 | 3 |
| 3 | 3 | 429 | 0 | 55 | 3 |
| 3 | 19 | 125 | 0 | 33 | 2 |
| 3 | 20 | 36 | 0 | 36 | 5 |
| 3 | 39 | 8 | 0 | 9 | - |
| 3 | 43 | 14 | 0 | 33 | - |
| 3 | 47 | 10 | 0 | 11 | 1 |
| 3 | 49 | 3 | 0.007 | 2 | - |
| 4 | 4 | 178 | 0 | 1 | 33 |
| 4 | 52 | 4 | 0.05 | - | - |
| 4 | 98 | 1 | 0.015 | - | - |
| 5 | 1 | 718 | 0 | 33 | 3 |
| 5 | 7 | 42 | 0.001 | 25 | - |
| 5 | 9 | 20 | 0.01 | 28 | - |
| 5 | 19 | 28 | 0 | 23 | 2 |
| 5 | 46 | 11 | 0 | 4 | 1 |
| 5 | 68 | 29 | 0 | - | - |
| 6 | 15 | 90 | 0 | 16 | - |
| 6 | 62 | 3 | 0 | 5 | - |
| 7 | 12 | 91 | 0 | - | 4 |
| 7 | 52 | 4 | 0.005 | - | - |
| 8 | 1 | 364 | 0 | 29 | - |
| 8 | 2 | 42 | 0 | 23 | - |
| 8 | 7 | 22 | 0.01 | 23 | - |
| 8 | 9 | 15 | 0 | 24 | 2 |
| 8 | 25 | 28 | 0 | 14 | - |
| 8 | 44 | 27 | 0 | 12 | - |
| 8 | 46 | 8 | 0 | 2 | - |
| 8 | 58 | 6 | 0 | 1 | - |
| 8 | 82 | 2 | 0 | - | - |
| 9 | 11 | 75 | 0 | 4 | - |
| 9 | 67 | 1 | 0.013 | 5 | - |
| 9 | 80 | 7 | 0 | 1 | - |
| 10 | 1 | 63 | 0 | 28 | - |
| 10 | 2 | 196 | 0 | 27 | 2 |
| 10 | 7 | 43 | 0 | 26 |  |
| 10 | 9 | 12 | 0.001 | 26 | 3 |
| 10 | 20 | 11 | 0.007 | 24 | - |
| 10 | 25 | 37 | 0 | 16 | - |
| 10 | 43 | 5 | 0.026 | 19 | - |
| 10 | 44 | 29 | 0 | 19 | - |
| 10 | 45 | 13 | 0 | 5 | - |
| 10 | 58 | 6 | 0 | 4 | - |
| 10 | 69 | 10 | 0 | 1 | 2 |
| 10 | 73 | 3 | 0 | - | - |
| 11 | 2 | 63 | 0.001 | 32 | - |
| 11 | 8 | 120 | 0 | 13 | 2 |
| 11 | 9 | 134 | 0 | 36 | 17 |
| 11 | 13 | 186 | 0 | 12 | 2 |
| 11 | 20 | 81 | 0 | 32 | 3 |
| 11 | 21 | 57 | 0 | 6 | 14 |
| 11 | 23 | 36 | 0 | 1 | 3 |
| 11 | 25 | 38 | 0 | 17 | 1 |
| 11 | 33 | 19 | 0 | 4 | - |
| 11 | 36 | 124 | 0.003 | 12 | 20 |
| 11 | 40 | 64 | 0 | 38 | 24 |
| 11 | 45 | 26 | 0 | 6 | 1 |
| 11 | 55 | 42 | 0.001 | 14 | 22 |
| 11 | 67 | 8 | 0 | 10 | 1 |
| 11 | 71 | 13 | 0 | - | - |
| 11 | 90 | 34 | 0 | - | - |
| 12 | 1 | 257 | 0 | 59 | - |
| 12 | 3 | 185 | 0 | 52 | - |
| 12 | 7 | 43 | 0 | 46 | 1 |
| 12 | 19 | 145 | 0 | 36 | - |
| 12 | 20 | 66 | 0 | 37 | - |
| 12 | 39 | 3 | 0.019 | 10 | - |
| 12 | 43 | 45 | 0 | 35 | - |
| 12 | 47 | 4 | 0 | 11 | - |
| 12 | 49 | 9 | 0 | 2 | - |
| 12 | 50 | 6 | 0.001 | 9 | - |
| 12 | 101 | 4 | 0 | 3 | - |
| 13 | 16 | 1 | 0.018 | 18 | - |
| 13 | 30 | 1 | 0.004 | 6 | - |
| 13 | 39 | 5 | 0 | 6 | - |
| 13 | 75 | 24 | 0 | 2 | - |
| 14 | 14 | 46 | 0 | - | - |
| 14 | 54 | 2 | 0 | - | - |
| 15 | 5 | 127 | 0 | 3 | 1 |
| 15 | 6 | 118 | 0 | 11 | 7 |
| 15 | 13 | 190 | 0 | 4 | 3 |
| 15 | 21 | 63 | 0 | 4 | 8 |
| 15 | 28 | 41 | 0 | - | 1 |
| 15 | 33 | 15 | 0.002 | 4 | 1 |
| 15 | 36 | 253 | 0 | 10 | 16 |
| 15 | 40 | 93 | 0 | 13 | 18 |
| 15 | 48 | 14 | 0 | 1 | - |
| 15 | 52 | 47 | 0 | 1 | - |
| 15 | 79 | 3 | 0.025 | - | 1 |
| 15 | 87 | 22 | 0 | 1 | 3 |
| 15 | 88 | 3 | 0 | - | - |
| 15 | 90 | 12 | 0.028 | - | 5 |
| 15 | 102 | 3 | 0.01 | 1 | - |
| 16 | 11 | 12 | 0 | - | - |
| 16 | 80 | 21 | 0 | 3 | - |
| 17 | 24 | 2 | 0.016 | - | - |
| 17 | 26 | 19 | 0 | - | - |
| 17 | 41 | 76 | 0 | - | - |
| 17 | 51 | 46 | 0 | - | - |
| 17 | 64 | 1 | 0.003 | - | - |
| 17 | 66 | 10 | 0 | - | - |
| 18 | 101 | 18 | 0 | - | - |
| 19 | 24 | 6 | 0 | - | - |
| 19 | 38 | 43 | 0 | 9 | 2 |
| 19 | 43 | 2 | 0.046 | 5 | - |
| 19 | 44 | 6 | 0 | 5 | - |
| 19 | 50 | 4 | 0 | - | - |
| 20 | 24 | 3 | 0 | - | - |
| 20 | 26 | 11 | 0 | - | - |
| 20 | 41 | 34 | 0 | 1 | - |
| 20 | 51 | 25 | 0 | 1 | - |
| 20 | 66 | 4 | 0 | - | - |
| 21 | 6 | 7 | 0.017 | 8 | 4 |
| 21 | 13 | 49 | 0 | 8 | 6 |
| 21 | 21 | 46 | 0 | - | 11 |
| 21 | 32 | 24 | 0 | 6 | 2 |
| 21 | 36 | 58 | 0 | 4 | 14 |
| 21 | 40 | 19 | 0 | 11 | 8 |
| 21 | 55 | 12 | 0 | 1 | 11 |
| 21 | 74 | 3 | 0.002 | - | 1 |
| 21 | 89 | 14 | 0 | 1 | 4 |
| 21 | 108 | 1 | 0.03 | - | 1 |
| 22 | 18 | 15 | 0 | 5 | 8 |
| 22 | 21 | 48 | 0 | 6 | 28 |
| 22 | 32 | 12 | 0 | 5 | 2 |
| 22 | 33 | 7 | 0 | 5 | 9 |
| 22 | 36 | 76 | 0 | 12 | 22 |
| 22 | 52 | 11 | 0.005 | 2 | - |
| 22 | 55 | 23 | 0 | 13 | 30 |
| 22 | 61 | 18 | 0 | - | 26 |
| 22 | 67 | 5 | 0 | 4 | 2 |
| 22 | 72 | 4 | 0.001 | 3 | 6 |
| 22 | 79 | 2 | 0.006 | - | - |
| 22 | 81 | 1 | 0.025 | - | - |
| 22 | 87 | 3 | 0.014 | 1 | 6 |
| 22 | 89 | 23 | 0 | 1 | 14 |
| 22 | 90 | 7 | 0 | - | - |
| 22 | 93 | 1 | 0.025 | - | 12 |
| 22 | 107 | 4 | 0 | - | 2 |
| 23 | 18 | 10 | 0 | 3 | 3 |
| 23 | 36 | 6 | 0.038 | 5 | 4 |
| 23 | 40 | 5 | 0.003 | 14 | - |
| 24 | 12 | 2 | 0.041 | - | - |
| 24 | 24 | 16 | 0 | 1 | - |
| 24 | 29 | 13 | 0 | 3 | 2 |
| 24 | 38 | 5 | 0 | 3 | - |
| 24 | 41 | 9 | 0 | - | - |
| 24 | 51 | 4 | 0 | - | - |
| 24 | 54 | 1 | 0.005 | 1 | - |
| 24 | 60 | 2 | 0 | - | - |
| 25 | 55 | 17 | 0 | 2 | 12 |
| 25 | 67 | 1 | 0 | 4 | - |
| 26 | 22 | 25 | 0 | 1 | - |
| 27 | 34 | 22 | 0 | 7 | - |
| 29 | 41 | 2 | 0.001 | - | - |
| 30 | 26 | 3 | 0 | - | - |
| 30 | 37 | 16 | 0 | - | 1 |
| 31 | 24 | 4 | 0 | - | - |
| 31 | 25 | 2 | 0.05 | - | - |
| 31 | 38 | 14 | 0 | - | - |
| 31 | 43 | 6 | 0 | - | - |
| 31 | 50 | 6 | 0 | - | - |
| 31 | 52 | 2 | 0.03 | - | - |
| 31 | 106 | 1 | 0.005 | - | - |
| 32 | 18 | 8 | 0 | - | 17 |
| 32 | 37 | 20 | 0 | - | 5 |
| 33 | 13 | 42 | 0.011 | 5 | - |
| 33 | 16 | 28 | 0 | 11 | - |
| 33 | 17 | 33 | 0 | 8 | - |
| 33 | 18 | 18 | 0 | 4 | 14 |
| 33 | 21 | 14 | 0.011 | 7 | 16 |
| 33 | 33 | 4 | 0.03 | 4 | - |
| 33 | 36 | 47 | 0 | 10 | 20 |
| 33 | 40 | 20 | 0.007 | 24 | 7 |
| 33 | 55 | 24 | 0 | 13 | 3 |
| 33 | 85 | 2 | 0.015 | - | - |
| 33 | 103 | 1 | 0.018 | - | - |
| 33 | 110 | 3 | 0.01 | - | 2 |
| 34 | 43 | 1 | 0.018 | - | - |
| 34 | 54 | 13 | 0 | - | - |
| 35 | 12 | 2 | 0.016 | 1 | 2 |
| 35 | 20 | 6 | 0 | - | 2 |
| 35 | 35 | 1 | 0.024 | - | 3 |
| 35 | 57 | 11 | 0 | - | 8 |
| 36 | 7 | 4 | 0.041 | 28 | - |
| 36 | 10 | 2 | 0.046 | 24 | - |
| 36 | 30 | 18 | 0 | 9 | 11 |
| 37 | 1 | 105 | 0 | 3 | - |
| 37 | 23 | 2 | 0.002 | - | - |
| 37 | 68 | 10 | 0 | - | - |
| 37 | 71 | 3 | 0 | - | - |
| 38 | 18 | 4 | 0.009 | 1 | 2 |
| 38 | 21 | 5 | 0.011 | 3 | 6 |
| 38 | 36 | 17 | 0.002 | 3 | 10 |
| 38 | 37 | 19 | 0 | 3 | 16 |
| 38 | 55 | 5 | 0.019 | 3 | 19 |
| 38 | 58 | 2 | 0.028 | 2 | - |
| 38 | 84 | 1 | 0.009 | - | 1 |
| 38 | 89 | 6 | 0 | - | 2 |
| 39 | 1 | 11 | 0.005 | 9 | - |
| 39 | 3 | 8 | 0 | 8 | - |
| 39 | 47 | 3 | 0 | 2 | - |
| 39 | 85 | 1 | 0 | 1 | - |
| 39 | 86 | 1 | 0 | - | - |
| 40 | 8 | 8 | 0 | 5 | 1 |
| 40 | 9 | 33 | 0 | 17 | 8 |
| 40 | 20 | 25 | 0 | 15 | 4 |
| 40 | 33 | 12 | 0 | 3 | 2 |
| 40 | 39 | 4 | 0 | 5 | 1 |
| 40 | 40 | 12 | 0.04 | 16 | 13 |
| 40 | 43 | 13 | 0 | 11 | 1 |
| 40 | 47 | 9 | 0 | 2 | 3 |
| 40 | 55 | 13 | 0 | 9 | 16 |
| 40 | 58 | 5 | 0.002 | 3 | - |
| 40 | 72 | 6 | 0 | 1 | 1 |
| 40 | 87 | 3 | 0.005 | 1 | 4 |
| 40 | 90 | 11 | 0 | - | - |
| 40 | 101 | 1 | 0.04 | - | - |
| 41 | 26 | 14 | 0 | - | - |
| 41 | 37 | 3 | 0 | - | - |
| 41 | 41 | 10 | 0 | - | - |
| 41 | 51 | 7 | 0 | - | - |
| 41 | 64 | 1 | 0 | - | - |
| 42 | 65 | 8 | 0 | 8 | - |
| 45 | 3 | 2 | 0.021 | - | - |
| 45 | 10 | 1 | 0.037 | - | - |
| 45 | 78 | 11 | 0 | - | 14 |
| 46 | 20 | 2 | 0.001 | - | 4 |
| 46 | 40 | 1 | 0.018 | 1 | 16 |
| 47 | 52 | 1 | 0.009 | - | - |
| 48 | 38 | 4 | 0 | - | - |
| 48 | 51 | 9 | 0 | 1 | - |
| 48 | 60 | 6 | 0 | - | - |
| 48 | 66 | 9 | 0 | - | - |
| 48 | 83 | 1 | 0.001 | - | - |
| 49 | 5 | 17 | 0 | 1 | - |
| 49 | 33 | 1 | 0.005 | - | - |
| 49 | 44 | 1 | 0.036 | - | - |
| 49 | 70 | 8 | 0 | - | 1 |
| 50 | 21 | 7 | 0 | 2 | 5 |
| 50 | 36 | 10 | 0.02 | 4 | 7 |
| 50 | 40 | 21 | 0 | 5 | 6 |
| 50 | 55 | 6 | 0.002 | 3 | 11 |
| 50 | 74 | 7 | 0 | - | 12 |
| 50 | 77 | 3 | 0 | - | 2 |
| 50 | 89 | 10 | 0 | - | - |
| 50 | 102 | 1 | 0 | - | - |
| 52 | 11 | 3 | 0 | - | - |
| 52 | 90 | 1 | 0.032 | - | - |
| 52 | 103 | 1 | 0 | - | - |
| 53 | 21 | 9 | 0 | - | 3 |
| 53 | 36 | 11 | 0 | - | 23 |
| 53 | 52 | 2 | 0.012 | - | 2 |
| 53 | 55 | 5 | 0.001 | - | 1 |
| 53 | 71 | 1 | 0.009 | - | - |
| 53 | 77 | 1 | 0 | - | - |
| 53 | 92 | 1 | 0 | - | 1 |
| 54 | 21 | 10 | 0 | - | 1 |
| 54 | 32 | 1 | 0.01 | 1 | - |
| 54 | 36 | 16 | 0 | 2 | 1 |
| 54 | 40 | 26 | 0 | 3 | 1 |
| 54 | 52 | 5 | 0 | - | - |
| 54 | 55 | 8 | 0 | - | 1 |
| 54 | 74 | 1 | 0.005 | - | - |
| 54 | 89 | 5 | 0 | - | 2 |
| 55 | 7 | 5 | 0.005 | 8 | - |
| 55 | 9 | 2 | 0.039 | 8 | 3 |
| 55 | 23 | 1 | 0.008 | - | 1 |
| 55 | 40 | 14 | 0 | 8 | 1 |
| 55 | 55 | 6 | 0 | 3 | 4 |
| 55 | 83 | 1 | 0 | - | - |
| 56 | 1 | 35 | 0 | 6 | - |
| 56 | 3 | 14 | 0 | 6 | 2 |
| 56 | 7 | 16 | 0 | 4 | - |
| 56 | 19 | 25 | 0 | 3 | 1 |
| 57 | 23 | 17 | 0 | - | 2 |
| 57 | 68 | 2 | 0.001 | - | - |
| 57 | 71 | 7 | 0 | - | - |
| 58 | 2 | 3 | 0.003 | 1 | - |
| 58 | 19 | 2 | 0.026 | 2 | - |
| 58 | 20 | 16 | 0 | 1 | - |
| 58 | 25 | 2 | 0.004 | 1 | - |
| 58 | 73 | 3 | 0 | - | - |
| 59 | 55 | 5 | 0 | - | - |
| 59 | 66 | 3 | 0 | - | - |
| 59 | 72 | 4 | 0 | - | - |
| 59 | 76 | 4 | 0 | - | - |
| 60 | 15 | 4 | 0 | 1 | - |
| 61 | 7 | 6 | 0 | 2 | - |
| 61 | 9 | 14 | 0 | 2 | 3 |
| 61 | 20 | 18 | 0 | 1 | - |
| 61 | 25 | 4 | 0 | 1 | 1 |
| 61 | 44 | 2 | 0.015 | 1 | - |
| 61 | 45 | 11 | 0 | 2 | - |
| 61 | 51 | 1 | 0.024 | - | - |
| 61 | 58 | 3 | 0 | 1 | - |
| 62 | 42 | 4 | 0 | 2 | - |
| 62 | 44 | 4 | 0 | 2 | - |
| 62 | 73 | 8 | 0 | - | 2 |
| 63 | 13 | 16 | 0 | - | - |
| 63 | 110 | 1 | 0.005 | - | - |
| 64 | 7 | 2 | 0.007 | - | - |
| 65 | 41 | 2 | 0.005 | 1 | - |
| 65 | 51 | 1 | 0.019 | 1 | - |
| 66 | 16 | 2 | 0 | 2 | - |
| 66 | 17 | 4 | 0 | 3 | - |
| 66 | 42 | 11 | 0 | 2 | - |
| 66 | 43 | 1 | 0.031 | 3 | - |
| 66 | 50 | 1 | 0.002 | 2 | - |
| 66 | 56 | 10 | 0 | 5 | - |
| 67 | 93 | 5 | 0 | - | 21 |
| 69 | 53 | 7 | 0 | - | 3 |
| 69 | 61 | 1 | 0.004 | - | 2 |
| 70 | 36 | 4 | 0.041 | 3 | 2 |
| 70 | 52 | 7 | 0 | 1 | - |
| 70 | 88 | 4 | 0 | - | 1 |
| 71 | 20 | 3 | 0 | 9 | - |
| 71 | 40 | 3 | 0 | 12 | - |
| 72 | 33 | 1 | 0.003 | - | 1 |
| 73 | 10 | 7 | 0 | 1 | - |
| 74 | 21 | 3 | 0 | - | - |
| 74 | 36 | 4 | 0.008 | - | - |
| 74 | 37 | 1 | 0.017 | - | - |
| 74 | 40 | 7 | 0 | - | - |
| 74 | 89 | 1 | 0.008 | - | - |
| 75 | 7 | 2 | 0.032 | - | - |
| 75 | 12 | 1 | 0.011 | 1 | - |
| 75 | 43 | 3 | 0 | - | - |
| 75 | 44 | 2 | 0 | - | - |
| 75 | 107 | 1 | 0 | - | - |
| 76 | 14 | 15 | 0 | - | 5 |
| 77 | 43 | 8 | 0 | - | - |
| 77 | 55 | 3 | 0.001 | - | - |
| 77 | 90 | 1 | 0.002 | - | - |
| 78 | 13 | 17 | 0 | - | - |
| 78 | 20 | 3 | 0 | 2 | 1 |
| 79 | 10 | 3 | 0 | 1 | - |
| 80 | 12 | 7 | 0 | 1 | 2 |
| 80 | 59 | 8 | 0 | - | 4 |
| 81 | 13 | 3 | 0.024 | 1 | - |
| 81 | 33 | 2 | 0 | - | - |
| 82 | 35 | 9 | 0 | 1 | 9 |
| 83 | 71 | 5 | 0 | - | - |
| 84 | 21 | 13 | 0 | - | - |
| 86 | 10 | 1 | 0.039 | - | - |
| 86 | 37 | 1 | 0.011 | - | 2 |
| 87 | 7 | 1 | 0.047 | - | - |
| 87 | 25 | 1 | 0.003 | - | 1 |
| 88 | 90 | 3 | 0 | - | - |
| 89 | 2 | 1 | 0.035 | 2 | - |
| 89 | 25 | 7 | 0 | 1 | - |
| 89 | 45 | 1 | 0 | 1 | - |
| 89 | 58 | 1 | 0.002 |  | - |
| 90 | 42 | 2 | 0 | 1 | - |
| 92 | 35 | 6 | 0 | - | 1 |
| 92 | 106 | 1 | 0.001 | - | - |
| 93 | 26 | 1 | 0.003 | - | - |
| 93 | 37 | 9 | 0 | - | 2 |
| 93 | 110 | 1 | 0.002 | - | - |
| 94 | 36 | 1 | 0.047 | - | - |
| 95 | 42 | 11 | 0 | 1 | - |
| 95 | 56 | 10 | 0 | 2 | - |
| 96 | 12 | 2 | 0.004 | - | - |
| 96 | 18 | 13 | 0 | - | 21 |
| 96 | 36 | 5 | 0.003 | - | 1 |
| 96 | 40 | 9 | 0 | - | 6 |
| 96 | 89 | 1 | 0.005 | - | 1 |
| 97 | 38 | 4 | 0 | 1 | - |
| 98 | 26 | 2 | 0 | - | - |
| 98 | 33 | 1 | 0 | 1 | - |
| 104 | 63 | 5 | 0 | - | 3 |
| 106 | 27 | 6 | 0 |  | - |
| 106 | 86 | 3 | 0 | 1 | - |
| 107 | 76 | 2 | 0 | - | - |
| 107 | 83 | 5 | 0 | - | - |
| 108 | 104 | 3 | 0 | 10 | - |
| 109 | 71 | 3 | 0 | 1 | - |
| 110 | 82 | 1 | 0 | - | - |
| 111 | 36 | 1 | 0.035 | - | - |
| 112 | 29 | 1 | 0 | 1 | - |
| 112 | 54 | 2 | 0 | - | - |
| 112 | 108 | 2 | 0 | 1 | - |
| 114 | 24 | 1 | 0 | - | - |
| 114 | 99 | 1 | 0 | - | - |
| 114 | 108 | 7 | 0 | - | - |
| 115 | 41 | 3 | 0 | - | - |
| 115 | 59 | 3 | 0 | - | - |
| 117 | 38 | 2 | 0 | - | - |
| 117 | 95 | 1 | 0 | 1 | - |
| 118 | 35 | 1 | 0.003 | - | - |
| 118 | 106 | 5 | 0 | - | - |

Note: P.value is used to measure the significance of crosstalk of each module pair of inflammation and cancer. – means none.
